# PCA and deep learning based myoelectric grasping control of a prosthetic hand

**DOI:** 10.1186/s12938-018-0539-8

**Published:** 2018-08-06

**Authors:** Chuanjiang Li, Jian Ren, Huaiqi Huang, Bin Wang, Yanfei Zhu, Huosheng Hu

**Affiliations:** 1 The College of Information, Mechanical and Electrical Engineering, Shanghai Normal University, Shanghai, 201418 China; 2 EPFL, 2002 Neuchâtel, Switzerland; 3 BFH, 2502 Biel, Switzerland; 4 School of Computer Science & Electronic Engineering, University of Essex, Colchester, CO4 3SQ UK

**Keywords:** Prosthetic hand, Grasp control, PCA, sEMG-force, DNN, Fuzzy controller, Vibration feedback device

## Abstract

**Background:**

For the functional control of prosthetic hand, it is insufficient to obtain only the motion pattern information. As far as practicality is concerned, the control of the prosthetic hand force is indispensable. The application value of prosthetic hand will be greatly improved if the stable grip of prosthetic hand can be achieved. To address this problem, in this study, a bio-signal control method for grasping control of a prosthetic hand is proposed to improve patient’s sense of using prosthetic hand and the thus improving the quality of life.

**Methods:**

A MYO gesture control armband is used to collect the surface electromyographic (sEMG) signals from the upper limb. The overlapping sliding window scheme are applied for data segmentation and the correlated features are extracted from each segmented data. Principal component analysis (PCA) methods are then deployed for dimension reduction. Deep neural network is used to generate sEMG-force regression model for force prediction at different levels. The predicted force values are input to a fuzzy controller for the grasping control of a prosthetic hand. A vibration feedback device is used to feed grasping force value back to patient’s arm to improve patient’s sense of using prosthetic hand and realize accurate grasping. To test the effectiveness of the scheme, 15 able-bodied subjects participated in the experiments.

**Results:**

The classification results indicated that 8-channel sEMG applying all four time-domain features, with PCA reduction from 32 to 8 dimensions results in the highest classification accuracy. Based on the experimental results from 15 participants, the average recognition rate is over 95%. On the other hand, from the statistical results of standard deviation, the between-subject variations ranges from 3.58 to 1.25%, proving that the robustness and stability of the proposed approach.

**Conclusions:**

The method proposed hereto control grasping power through the patient’s own sEMG signal, which achieves a high recognition rate to improve the success rate of grip and increases the sense of operation and also brings the gospel for upper extremity amputation patients.

## Background

Hand amputation is a dramatic event for human being and will greatly degrade the life quality of amputees. It was estimated that there were three million upper limb amputees all over the world in 2016 [1]. The major causes of hand amputations include trauma, dysvascularity, and neoplasia. Depending on the amputation level, upper limb amputation can be classified into partial hand amputation, wrist disarticulation, transradial (below elbow), transhumeral (above elbow), and shoulder disarticulation.

A hand or arm prosthesis is a device that aims to replace the missing hands or arms. About 80% of the upper limb amputees are reported to use some types of prostheses [2]. The upper limb prostheses can be categorized into passive prostheses and active prostheses. Passive prostheses mainly serve an aesthetic purpose and sometimes help to balance the body in order to avoid spinal misalignment. Active prostheses can provide grasping functions. Within the active prostheses, the body-powered (BP) prostheses can be open or closed through a harness and cable system worn on the shoulder. They are simple to use, robust, and inexpensive. However, their drawbacks include the high expenditure of energy required from the user and the limited control interface since most BP prostheses only offer one degree-of-freedom (DOF) at a time. The other type of active prostheses is electrically powered ones.

Many of the electrically powered prostheses are controlled by sEMG signals and are referred as myoelectric prostheses. sEMG signals measure the muscle generated electrical currents during contraction [3], which represents neuromuscular activities [4]. Commercial myoelectric prostheses generally apply two sEMG electrodes over the flexor and extensor muscles of the forearm for transradial amputees or over the biceps and triceps for transhumeral amputees or over the pectoralis and deltoid for shoulder disarticulation amputees. In research, the number of sEMG electrodes used ranges from 2 to 32. The myoelectric prostheses provide the most dexterous and intuitive control [5].

**Fig. 1:**
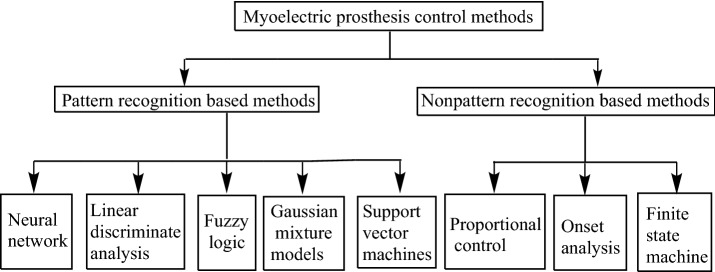
Classifications of myoelectric control methods

In general, myoelectric prosthetic control methods can be divided into two types, namely pattern recognition and non-pattern recognition methods, as shown in Fig. 1. The pattern recognition based methods include neural networks, linear discriminate analysis, fuzzy logic, Gaussian mixture models and support vector machines. In contrast, the non-pattern recognition based methods include proportional control, onset analysis and finite state machines. For hook-like myoelectric prostheses (1 DOF), the on–off control and the proportional control are generally used to open or close the hand. For on–off control, muscle flexion closes the hand and muscle extension opens the hand, or vice versa. On top of on–off control, proportional control adds speed or force control: the opening and closing of the prosthetic hand is proportional to the magnitude of the sEMG signal. For multi-grasp prostheses, finite-state-machine (FSM) control is widely used in commercial prostheses (e.g. i-Limb [6]) as well as in some research hands (e.g. southampton REMEDI hand [7]). Several sets of states are first defined, and the muscle flexion and closing change within each state. The muscle contractions switch between the states in a predefined sequential order. Although FSM control can achieve many grasping types, its intrinsic sequential control requires a number of actions before reaching the desired state.

Pattern recognition based control methods apply machine learning algorithms to detect and classify certain patterns of sEMG signals. The pattern recognition based control consists of four steps: data acquisition, data segmentation, feature extraction, and classification. Pattern recognition can be intuitive when the muscle contraction that would generate a movement in the unimpaired limb is mapped to the same movement in the prosthesis. Many machine learning algorithms have been proposed for sEMG pattern recognition: neural network [8, 9], linear regression analysis [10, 11], and support vector machine [11], to name a few. For most of pattern recognition based methods, machine learning is used for pattern classifications of hand postures. However, for precise grasping control, the grasping force prediction is essential. To the best of our knowledge, only limited research has been attempted to predict grasping force levels using machine learning algorithms [12, 13].

In this paper, we propose a new control method for grasping control of a prosthetic hand based on PCA and DNN. “Methods” section describes the system design, feature abstraction, dimension reduction, and pattern recognition. More specifically, PCA is deployed to reduce dimension of time domain (TD) feature matrix, predicting grasping strength by establishing regression model using DNN. In “Results” section, experimental results are presented to show the feasibility and good performance of the proposed approach, including the classification accuracy concerning different sEMG channel configurations, different feature combinations, and different degrees of dimension reduction. In "Discussion" section, the method is further analyzed and the rationality of the scheme is confirmed. Finally, a brief conclusion and future work are given in “Conclusions and future work” section.

## Methods

### The system design

**Fig. 2:**
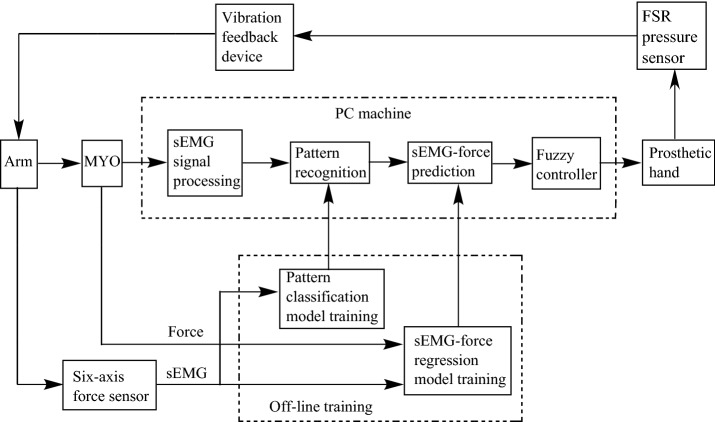
The proposed approach for griping control of a prosthetic hand

**Fig. 3:**
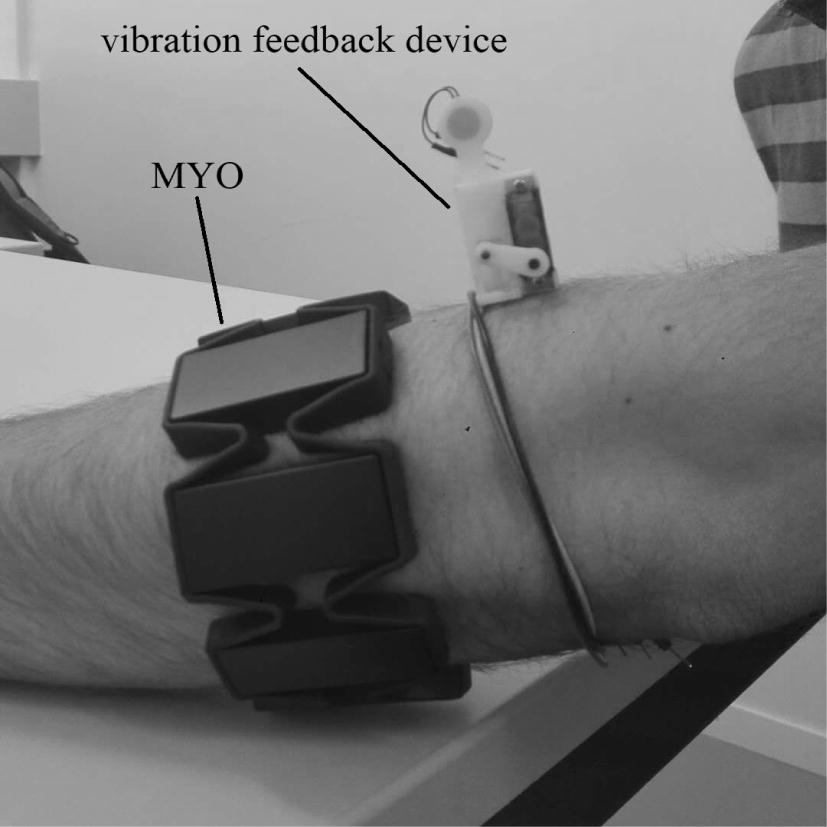
sEMG and force signal acquisition

**Fig. 4:**
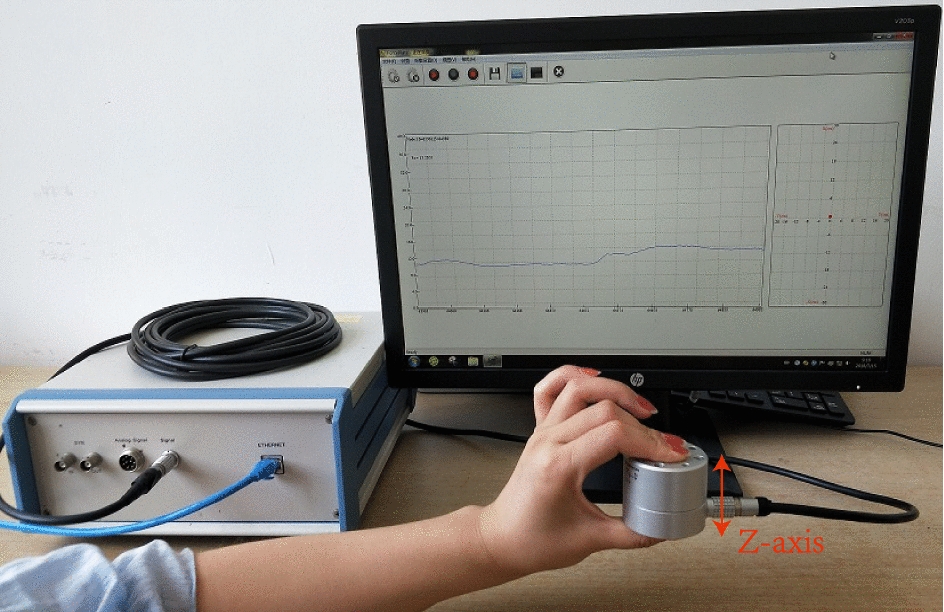
Grasping force signal obtain for training data

This paper aims at using deep neural network to classify grasping force levels. As people use three-finger pinch gesture most in daily life, and three-finger pinch can complete most of the objects grasp, the grip research of this paper is mainly based on three-finger pinch. Figure 2 shows the schematic of the proposed approach for prosthetic hand griping control. The proposed system consists of a MYO (shown in Fig. 3), used to collect sEMG signals of upper limb, and a six-axis force sensor [14] (as shown in Fig. 4), deployed to measure the corresponding grasping force of upper limb. The rest of the section focuses on the experimental setup, subjects’ demographics, the feature extraction and reduction algorithms, and the DNN architecture. For grasping force control, we divide force into different levels, set the predicted force value as the given signal, use fuzzy control to control grip strength of prosthetic hand. And for the sake of improving patient’s sense of using prosthetic hand and realize accurate grasping, a force-sensitive resistor (FSR) is fixed on the pad of finger to gain the exact amount of force applied by the system, a vibration feedback device to feed force level value back to patient’s arm is adopted. The calculation of TD features is simple, in addition, PCA further reduces the amount of computation and shortens calculation time. TD features extraction and PCA dimension reduction could realize the real-time control of prosthesis grasping. The regression model established by DNN is simple and could obtain good prediction results. A fuzzy controller is used to control the grasping force of a prosthesis hand and a vibration feedback device (shown in Fig. 3) to feedback force value to patient’s arm can improve the patient’s sense of using prosthetic hand.

### Features extraction

In order to distinguish and collect sEMG signals under different grasping strength, grasping force is evenly divided into 8 levels between 0 and 40 N (level 1: 0–5 N, level 2: 5–10 N, and so on). Most of objects can be grasped by dividing grip strength into different grades as the use of continuous values for grasping control may make the grip process unstable. Additionally, vibration feedback can only give several levels of grip force feedback which cannot meet the demand of grasping control. Due to the particularity of three-finger pinch, this paper mainly considers the force in the Z-axis direction (as shown in Fig. 4) when collecting the force signal. Participants apply force to the Z-axis of the sensor with a three-finger pinch gesture at different force levels so that grasping force signals could be obtained.

According to the TD feature of sEMG, we adopt a specific method to calculate the sum of mean absolute value (MAV) of 8 channels sEMG, and then compare with the preset threshold value to judge the starting point and the ending point of action, i.e. to determine starting and ending time of movement.

As the accuracy of force prediction is related to the sEMG features, it is important to select suitable features from the commonly used sEMG features such as TD features, frequency domain (FD) features and other more complex features [15–17]. In this study, four representative TD features are selected: MAV, root mean square (RMS), SD, and waveform length (WL). MAV contains the information of the average intensity and the concentration of the sEMG signal. In the process of the movement, RMS represents the contribution of each muscle organization. SD can demonstrate the dispersion degree of a data set. WL reflects the complexity of sEMG waveform and the joint effect of sEMG amplitude, frequency and duration [18]. TD calculation is simple, and thus can ensure real-time grasping [19]. The aforementioned features can be calculated from:

1 $$\begin{aligned} MA{V_k} = \frac{1}{N}\sum \limits _{i = 1}^N {\left| {x(i)} \right| }. \end{aligned}$$

2 $$\begin{aligned} RM{S_k} = \sqrt{\frac{1}{N}\sum \limits _{i = 1}^N {x{{(i)}^2}} }. \end{aligned}$$

3 $$\begin{aligned} S{D_k} = \sqrt{\frac{1}{{N - 1}}\sum \limits _{i = 1}^N {{{(x(i) - \mu )}^2}} }. \end{aligned}$$

4 $$\begin{aligned} W{L_k} = \sum \limits _{i = 1}^{N - 1} {\left| {x(i + 1) - x(i)} \right| }. \end{aligned}$$

where *x*(*i*) is the sEMG data of each sample, $\mu$ is the average of *N* data, $k=1,\ldots ,M$, with *M* being the number of channels. Since the extraction of each feature value in this paper is based on overlapping sliding window, *N* is the size of sliding window. In this design, window size is set to 50 ms, and sliding increment is set to 25 ms. As for force signal, use the same window settings. And extract the mean value of the data in the window as the feature of the sEMG signal.

### PCA dimension reduction

Considering the real-time requirements of the control system, PCA dimension reduction technique is used to reduce the computational complexity, shorten the computing time, and improve the grasping speed [20]. A section of sEMG is intercepted as a 1-dimensional signal sequence for a complete hand movement. Assuming that the number of the feature extracted is *n*, the dimension of the feature vector is $1 \times n$, and $U\in R^{n \times k}$ is the dimension reduction matrix by PCA. The feature matrix multiplied by the dimension reduction matrix becomes $1 \times k$, i.e. reducing from the original n-dimension down to k-dimension $(k < n)$. PCA can be used to analyze the main influencing factors from multiple sources and simplify complex problems [21].

### Pattern recognition and force prediction based on DNN

A standard ANN consists of three layers: an input layer, a hidden layer, and an output layer. A mathematical representation of a neural network is the propagation of function from input layer to the output layer:

5 $$\begin{aligned} u = f(\phi (w,x)). \end{aligned}$$

where *x* and *u* are the input and output of an artificial neural, *w* is the corresponding weight of the link between the input and the transfer function. $\phi (w,x)$ normally takes the linear combination of *w* and *x*, $f(\cdot )$ is the transfer function, which can take many forms, the most-widely used ones including linear function, step function, and sigmoid function.

Besides a transfer function, an ANN is characterized by two other parameters, namely the link pattern and the weight of each link, which are pre-determined. The weight of each link needs to be trained using the training data. Back-propagation is commonly used in ANN training due to its fast convergence. It consists of two steps: propagation and weigh update.

In the current study, DNN is applied for classifying the force levels, which is a type of ANN with multiple hidden layers. Compared to conventional ANN (with one hidden layer), DNN can represent a complex non-linear model. In the current study, we have applied a DNN with a two-layered stacked auto-encoder and a softmax classifier as the output layer. Each hidden layer consists of 200 hidden neurons. The desired average activation of the hidden units is 0.1. The weight of decay parameter is donated as $\lambda =3\times 10^{-3}$. The weight of sparsity penalty term is donated as $\beta =1$.

The training process consists of two steps: (i) the weights of the hidden layers are trained using unlabeled data through a greedy layer-wise approach, and the softmax classifier is trained using labeled data to map the inputs to assigned levels. (ii) The network is fine-tuned using labelled data through backpropagation.

### Subjects description and experimental procedure

**Fig. 5:**
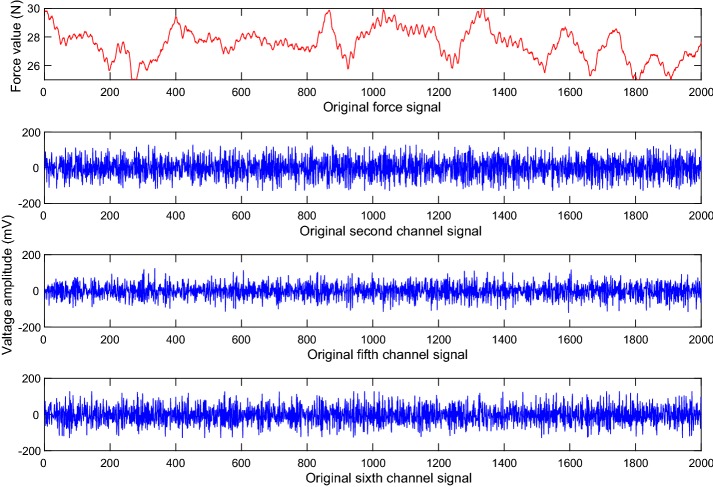
sEMG original waveform signals and grasping force waveform for force level 6

Fifteen able-bodied subjects (8 males and 7 females) participate in this experiment, ages range from 20 to 30. The sEMG signal were collected through a MYO (8 channels), sampled at 200 Hz per channel. The force signal were collected using a six-axis force sensor, sampled at 200 Hz. Figure 5 shows a random selected original sEMG waveform and a grasping force waveform for the force level 6 (25–30 N).

Firstly, to reduce skin impedance and improve sEMG signal quality, body hair removal and cleaning with alcohol are performed to the collecting parts of participants’ arms. A MYO was used to collect the sEMG signals. A six-axis force sensor was used to collect grasping force signals. Participants wore the MYO and pinched with the six-axis force sensor with 8 different levels force. For each level, participants kept pinch motion for four to 6 s; then returned to relaxed state, and after ten seconds repeated the hand motion. Each participant repeated the steps above ten times. Between each 2 levels of force, rest 20 min. The method above is used to collect training signal. The testing signal acquisition method is consistent with the training signal acquisition method, but there should be one hour break between training signal and testing signal collection.

## Results

The collected sEMG data was first segmented using the overlapping sliding window scheme. The feature or combined features were extracted within each window. Then PCA was used to reduce the feature dimensions. By using 400 groups of training data and 400 groups of testing data for each of the 8 levels of force, we have 6400 groups of data for each participant in total. 200 groups of training data randomly selected among each level are used for training, and 200 groups of testing data randomly selected among each level are used for prediction.

Each group of training data consists of eight sEMG features and one force feature. Eight sEMG features are selected as input and one force feature is selected as output of DNN. Similarly, each group of validation data consists of eight sEMG features and one force feature. Eight sEMG features are selected as input to produce an actual output value by DNN. But the one force feature is selected as theoretical output value to compare with actual output value to obtain recognition accuracy and prediction results.

**Fig. 6:**
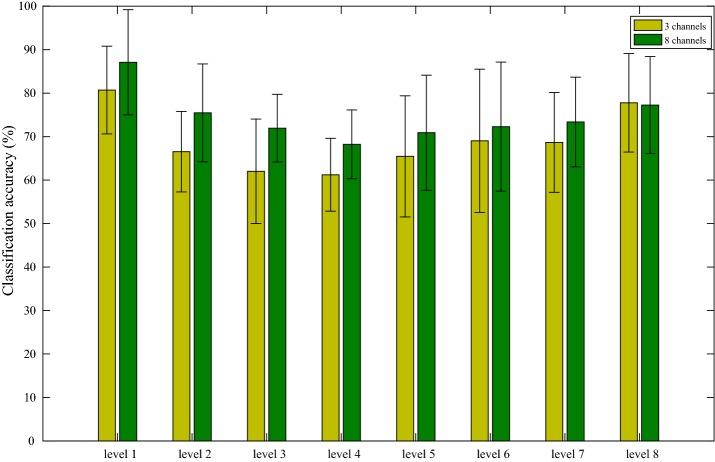
The average classification accuracy of 3 and 8 channels in classifying 8 force levels. The error bars represent the SD value over 15 subjects. The X-axis represents the forces at 8 different levels. The Y-axis represents average recognition rate (%) over 15 participates. The X-axis and Y-axis in Figs. 7 and 8 have the same meaning

In order to reduce the amount of calculation, ensure real-time control, and improve force prediction accuracy, we compared and analyzed the classification accuracy of 8 levels of force among different channels of sEMG, different features, and PCA dimension reduction. We observed that 3 channels were fluctuated obviously by mapping 8 channels of sEMG signal collected, and they were relatively sensitive and ideal for the grasping movement. Figure 6 shows the prediction results of sEMG signals from 3 and 8 channels, respectively. As can be seen, by extracting four TD features, and their 8 levels force pattern recognition, the classification accuracy for 3 channels is slightly inferior to that of 8 channels and therefore only the 8 channels scenarios are considered in the following processing. From the error bars, the value of SD is very large, which means the interpersonal prediction results are quite different and this scheme has a very low robustness.

**Fig. 7:**
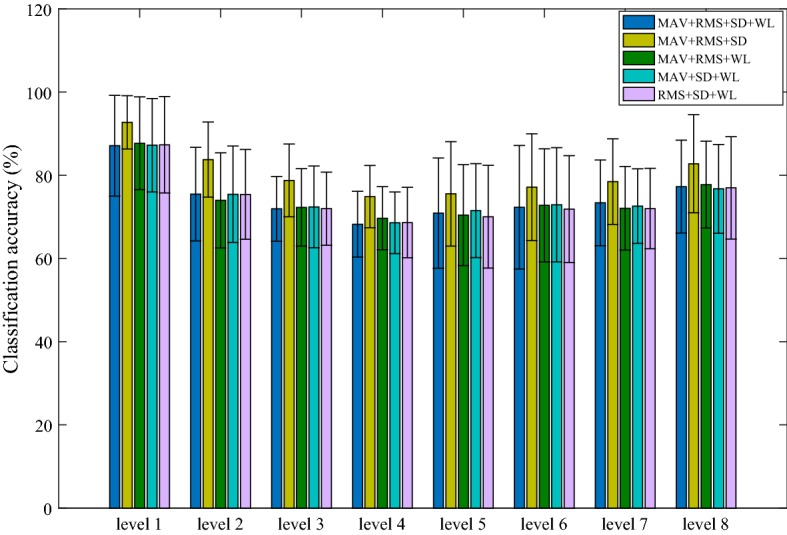
The average classification accuracy of different TD feature combination in classifying 8 force levels. The error bars represent the SD value over 15 subjects

Figure 7 shows the results of four features and a combination of three features obtained from the 8 channels of sEMG signals. As can be seen, the selection and number of features have little effect on recognition rate and SD value for the 8 channels of sEMG signal. Therefore, four eigenvalues were extracted for each channel signal within the subsequent processing.

**Fig. 8:**
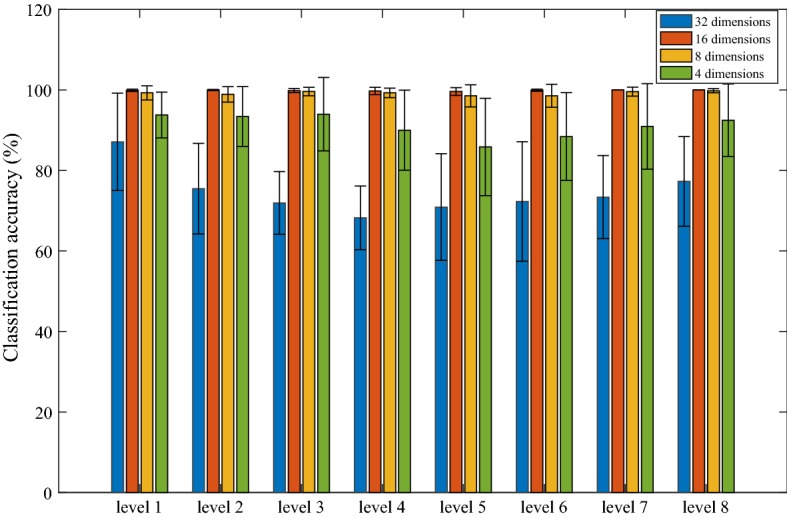
Pattern recognition rate of 8 channels with four TD features reduced by PCA to different dimensions. The error bars represent the SD value over 15 subjects

For the 8 channels of sEMG, four features are extracted from each channel. A feature matrix with 32-dimension is created, which is then reduced by PCA to 16, 8, or 4 dimensions. The results of the 4 dimension reduction cases are shown in Fig. 8. It shows that the recognition rates of 16 and 8 dimensions are better than that of 32 and 4 dimensions in the experiment. Moreover, the value of SD shows the same trend. In addition, the time of reducing dimension from 32 to 16 or 8 is the same, but the amount of calculation of 16 dimensions is much larger than that of 8 dimensions. Therefore, we choose the method of reducing dimension from 32 to 8, which sacrifices a little bit of recognition rate in exchange for real-time performance of grasping.

In the previous preparations, we did comparison among different window sizes, such as 50, 100, 200, 250, 300, 400, 600, 800 ms, and 1 s. But with the influence of PCA, different window size has little effect on the recognition rate, so it’s not listed one by one here.

**Fig. 9:**
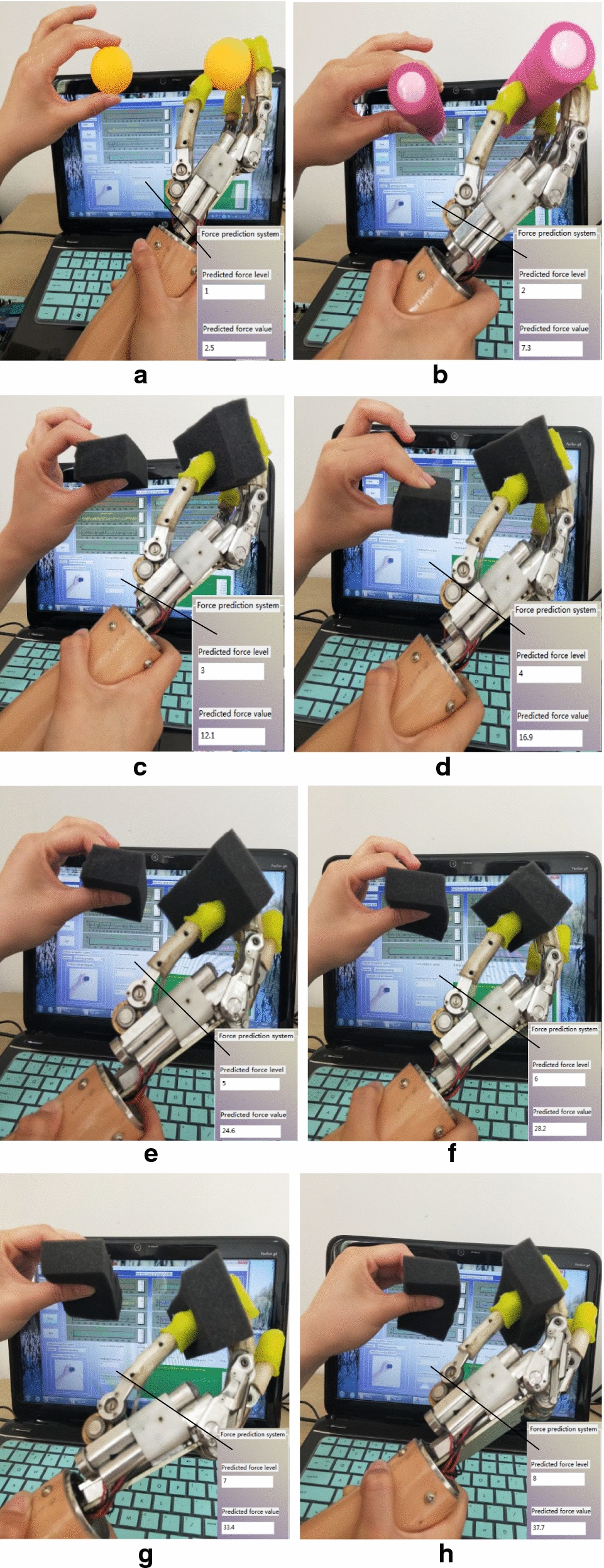
Use different levels of force to grasp different objects. **a** The force level 1, **b** shows the force level 2. The shape change of sponge with different force levels: **c** shows the force level 3, **d** shows the force level 4, **e** shows the force level 5, **f** shows the force level 6, **g** shows the force level 7, **h** shows the force level 8

During the implementation of the proposed approach, individual participants use different levels of force to control the prosthetic hand to grasp different things in Fig. 9. Figure 9a shows the results of grasping a table tennis with force level 1, and Fig. 9b shows the results of grasping a cylinder with force level 2. As can be seen in Fig. 9, the sponges have different degrees of deformation under different force levels. Because the last few force levels are relatively large, it is possible that the deformation of the sponge cannot be clearly seen by the naked eye. The personal computer (PC) interface shows the force level of participant and at the same time, the prosthetic hand receives a grip order to grasp the object by corresponding force level. Finally, the participants adjust their grip strength according to signals from a FSR pressure sensor and a vibration feedback device as described above.

## Discussion

In this paper we proposed the method based on PCA and DNN to achieve grasping control of prosthetic hand. The sEMG signal and force signal of 15 people were collected to build sEMG-force regression model. In order to realize stable grasping control, we divided force into different levels. Further, we analyzed the average classification accuracy of different circumstances in classifying 8 force levels, including 3 channels sEMG and 8 channels sEMG, four TD features and different three TD features combination, 32, 16, 8 and 4 dimensions feature matrix. From experimental results, 8 channels sEMG contains more information than 3 channels sEMG to have a better classification accuracy. TD feature has a good effect on classifying 8 force levels, and the results of different combinations are not very different. PCA plays a very important role in this paper. With the help of PCA, we get a better result when dimension reduced to 8. On the other hand, it reduces the amount of computation and provides real-time conditions for grasping the prosthetic hand.

The above method for processing myoelectric signals and features, we used post hoc analysis. therefore, it’s essential to conduct a general analysis of the above-mentioned methods. Table 1 shows the SD values of each case over 15 subjects. Among them, 32 dimension also stands for the case of 8 dimensions and TD feature combination of MAV, RMS, SD and WL (i.e. MAV + RMS + SD + WL). From the statistical results of the table, in each case, the recognition results of everyone’s 8 different levels are not much different. Especially for the case of 8 dimensions, the maximum value of SD is 3.58% and the minimum value of SD is 1.25%. Both values are small, which stands that the results of these 15 individuals are very similar and verifies that the approach used is universal. Secondly, The study provided the basis for other people or our own follow-up study of this issue. Others can use this conclusion directly without the need for post-mortem analysis.

**Table 1:** The SD values of each case over 15 subjects

	Level 1 (%)	Level 2 (%)	Level 3 (%)	Level 4 (%)	Level 5 (%)	Level 6 (%)	Level 7 (%)	Level 8 (%)
3 channels	13.23	8.57	12.26	10.22	12.57	13.95	12.47	9.61
MAV + RMS + SD	10.88	10.59	6.50	5.08	13.51	14.69	12.06	7.25
MAV + RMS + WL	12.47	8.80	6.90	6.33	12.21	12.33	13.07	7.87
MAV + SD + WL	14.40	10.52	6.51	6.08	10.41	13.43	11.91	8.39
RMS + SD + WL	12.72	8.68	5.59	8.89	10.98	13.89	10.63	9.92
32 dimensions	12.50	9.80	10.46	8.27	11.85	14.09	12.86	9.54
16 channels	0.41	0.49	1.25	1.41	1.05	0.00	0.26	0.00
8 channels	2.53	3.58	2.54	2.61	2.72	1.79	2.09	1.25
4 channels	5.76	7.04	8.16	10.52	5.15	9.15	8.93	11.80

**Fig. 10:**
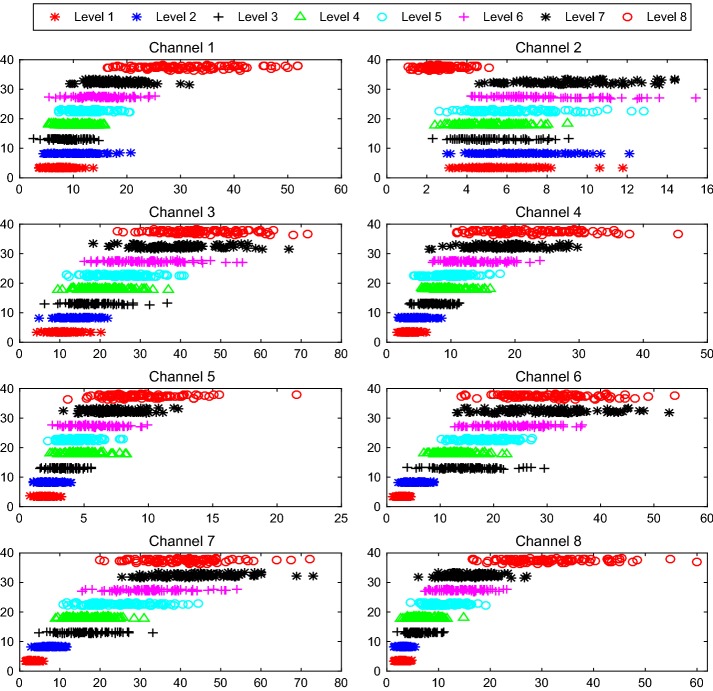
Dispersion and clustering of MAV under 8 force levels. The X-axis represents the value of features from channel 1 to channel 8. The Y-axis represents the value of 8 force levels. The X-axis and Y-axis in Figs. 11, 12, and 13 have the same meaning

**Fig. 11:**
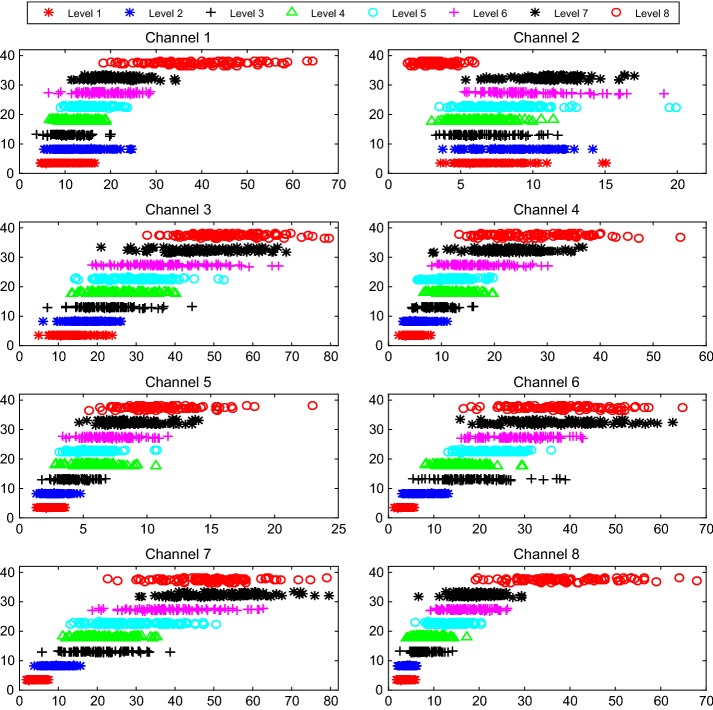
Dispersion and clustering of RMS under 8 force levels

**Fig. 12:**
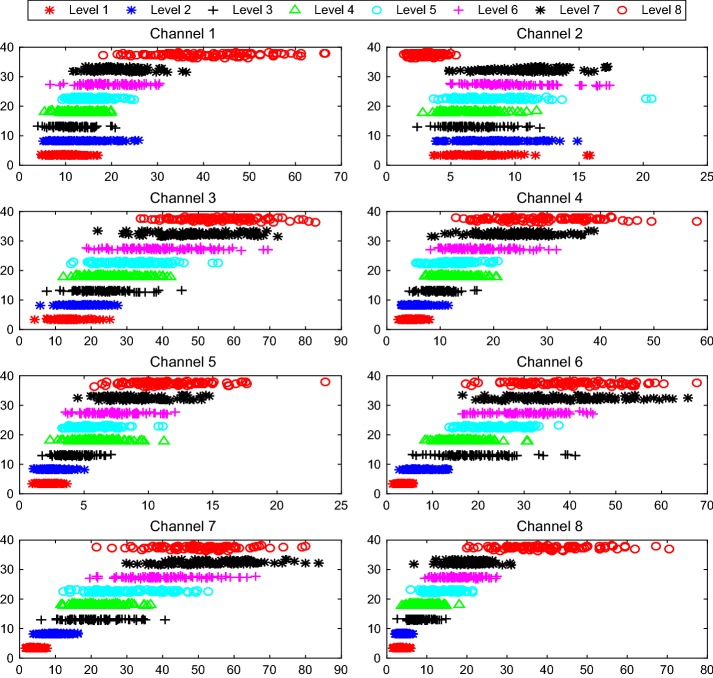
Dispersion and clustering of SD under 8 force levels

**Fig. 13:**
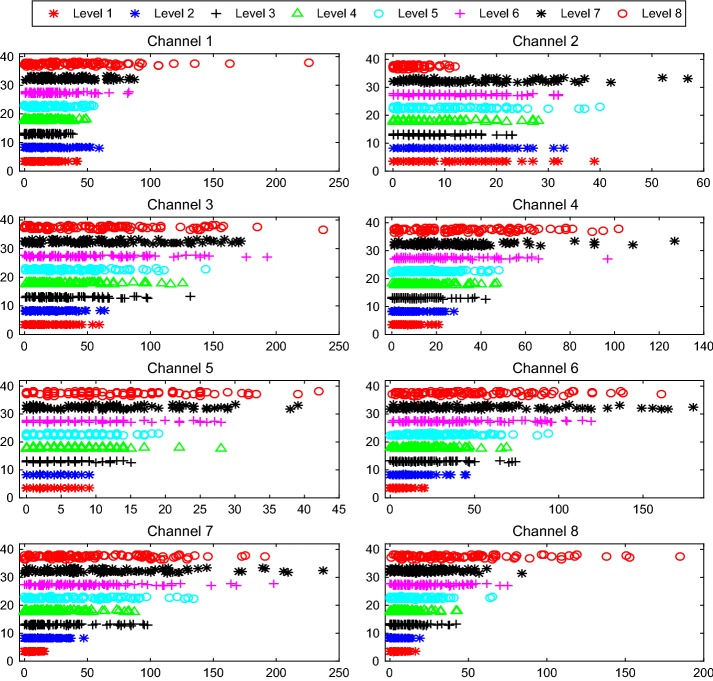
Dispersion and clustering of WL under 8 force levels

To test and verify the four TD features selected have enough variation with force to construct a good classifier, we analyzed one person’s dispersion and clustering of each feature vs. force. And the results show in Figs. 10, 11, 12, and 13. From which we can see that the 8 force levels have a good degree of differentiation. Therefore, we can conclude the four TD features selected and the approach are suitable.

As for grasping force control, the signal is collected from one healthy subject, which includes 8 levels of grasping force collected from a six-axis force sensor, and 8 channels sEMG signal gathered from MYO corresponding to 8 levels. Four TD features were abstracted into a feature matrix used for training pattern classification model. The feature matrix with force data are then used to construct sample for sEMG-force regression model training. The parameters of each model is saved and written to PC. After the preparation, each participant can wear MYO to collect and process sEMG over a PC machine. Then pattern classification model was used to recognize which level the sEMG signal belongs to, and the corresponding level of sEMG-force model was used to predict force value.

What follows is that the predicted force value was used as the given signal to control grasping force of a prosthetic hand. The grasping strength of the prosthetic hand was induced by using the FSR pressure sensor fixed on the prosthetic hand. Then vibration feedback device was matching with the force level value, and feedback to the participant. Different force levels were converted into different vibration frequencies in order to make patient feel the real grasping value and adjust grip strength accordingly for good accuracy.

## Conclusions and future work

In this paper, we proposed a novel approach to myoelectric grasping control of a prosthetic hand based on PCA and deep learning. PCA is used to reduce dimension of TD feature matrix, predicting grasping strength by establishing regression model between sEMG and force. PCA dimension reduction for sEMG signal provides the real-time control of prosthesis grasping. The regression model established by DNN is simple and has a good prediction effect. Adding the vibration feedback device to indicate the applied force value back to participant’s arm can improve the patient’s sense of using the prosthetic hand. The experimental results show the feasibility, robustness and good performance of the proposed approach.

The future work will focus on the adaptation of this system for amputees in order to test the feasibility and performance of the system and provide indicators for subsequent improvements. We will also compare the experimental results performed on forearm amputees with those performed on healthy subjects for further improvements.

## Abbreviations

MYO: MYO gesture control armband
sEMG: surface electromyogram
PCA: principal component analysis
DNN: deep neural network
TD: time domain
SD: standard deviation
BP: body-powered
DOF: degree of freedom
FSM: finite-state-machine
MAV: mean absolute value
FD: frequency domain
RMS: root mean square
WL: waveform length
ANN: artificial neural network
PC: personal computer
FSR: force-sensitive resistor
